# Expression Pattern of Angiogenic Factors in Healthy Heart in Response to Physical Exercise Intensity

**DOI:** 10.3389/fphys.2019.00238

**Published:** 2019-03-28

**Authors:** Marianna Bellafiore, Giuseppe Battaglia, Antonino Bianco, Antonio Palma

**Affiliations:** Department of Psychology, Educational Science and Human Movement, University of Palermo, Palermo, Italy

**Keywords:** heart, skeletal muscle, endurance training, angiogenesis, physical exercise, capillary growth, hypoxia, exercise intensity

## Abstract

Recently, many studies showing the regeneration potential of both cardiac and hematopoietic stem cells in adult heart following injury were definitively retracted by the literature. Therefore, stimulating myocardial angiogenesis becomes to be important for preventing cardiovascular diseases. Regular endurance exercise has been reported to induce capillary growth in healthy and diseased myocardium resulting in cardioprotective phenotype. Previously, we demonstrated a significantly increased capillary proliferation in mouse hearts following 30 and 45 days of endurance training. In the present study, we examined the localization and expression pattern of vascular endothelial growth factor receptors (VEGFR-1/Flt-1 and VEGFR-2/Flk-1), hypoxia-inducible factor-1α (HIF-1α), and inducible nitric oxide synthase (iNOS) in heart neocapillarization in response to a mild, moderate, and high intensity of endurance training. Sixty-three Swiss male mice were divided into four untrained control groups and three groups trained for 15 (T15), 30 (T30), and 45 (T45) days with a gradually increasing intensity on a treadmill. We observed the localization of studied proteins with immunostaining and their expression level with Western blot analyses. We found that VEGFR-2/Flk-1 expression progressively increased in trained groups compared with controls, while VEGFR-1/Flt-1 and HIF-1α were higher in T15 than in controls, T30, and T45 animals. Differently, iNOS levels enhanced after 15 and 30 days of exercise. The localization of these factors was not altered by exercise. The results showed that the expression of VEGFR-1/Flt-1, VEGFR-2/Flk-1, HIF-1α, and iNOS is differently regulated in cardiac angiogenesis according to the exercise intensity. VEGFR-1/Flt-1 and HIF-1α are upregulated by a mild intensity exercise, while VEGFR-2/Flk-1 progressively enhances with increasing workload. Differently, iNOS protein is modulated by a moderate intensity exercise. VEGF pathway appears to be involved in exercise-related angiogenesis in heart and VEGF might act in a paracrine and endocrine manner. Understanding this relationship is important for developing exercise strategies to protect the heart by insults.

## Introduction

Regular physical exercise is well known to reduce cardiovascular diseases, the main cause of death worldwide, increasing cardiac function and protecting against myocardial damage (Schuler et al., 2013; Tao et al., 2015). Heart adaptations can vary according to type, intensity, and duration of exercise and are commonly defined as the athlete’s heart (Pluim et al., 2000; Fulghum and Hill, 2018). For instance, endurance exercises such as running or swimming performed for prolonged periods are a significant physiological stimulus for an enhanced demand of oxygen and nutrient leading to the formation of new capillary in myocardium (Brown, 2003; Bellafiore et al., 2007; Fulghum and Hill, 2018). In detail, modifications in the blood flow, muscle contraction, and oxygen levels associated with hemodynamic mechanical events have been shown to be key signals for triggering vessel wall remodeling and activation of growth factors involved in the proliferation, migration, and tube formation of endothelial cells in both heart and skeletal muscle (Brown, 2003; Prior et al., 2004). This implies a cross talk between skeletal and cardiac muscle in order to respond synergistically to various stimuli derived by physical exercise. Indeed, several studies recently reported that exercise stimulates the release of circulating cytokines and growth factors, called myokines, by skeletal muscle that acts with endocrine functions mediating exercise-induced cardiovascular adaptations (Giudice and Taylor, 2017; Hoffmann and Weigert, 2017).

Among the molecules involved in the angiogenesis induced by physical exercise, vascular endothelial growth factor (VEGF) plays a crucial role in the induction of endothelial cell mitosis and promotion of capillary sprouting (Hudlicka et al., 1995; Shibuya, 2006). The angiogenic action of VEGF is mediated by two primary receptors, such as VEGFR-1/Flt-1 and VEGFR-2/Flk-1, both predominantly expressed on the endothelial cells but with different roles (Hudlicka et al., 1995; Iemitsu et al., 2006; Shibuya, 2006). VEGFR-1/Flt-1 has higher affinity for VEGF than VEGFR-2/Flk-1, and it was found to mediate chemotaxis, mitogenesis, and cytoskeletal reorganizations. In contrast, VEGFR-2/Flk-1 has a ligand-induced autophosphorylation activity much stronger than VEGFR-1/Flt-1 and is the major regulator of vasculogenesis and angiogenesis (Shibuya, 2006). Iemitsu et al. (2006) found an increase in mRNA and protein expression of VEGFR-1/Flt-1 and VEGFR-2/Flk-1 associated with an enhanced capillary density in aged rat hearts after 8 weeks of swim training. Furthermore, Milkiewicz et al. (2003) showed that these receptors were regulated by chronic ischemia/hypoxia and intermittent electrical stimulation in rat skeletal muscle. The role of hypoxia as a candidate for the initiation of angiogenesis in exercising muscle was reinforced by data indicating that hypoxia-inducible factor-1 α (HIF-1α) expression induced by hypoxia determined the upregulation of VEGF in trained rat muscles (Shweiki et al., 1992). Moreover, HIF-1α has been found to be significantly overexpressed in rat left ventricle 48 h after the last training session of a 10-week mild intensity aerobic exercise protocol and to participate in cardioprotection (Giusti et al., 2009). This effect might be due to “ischemic preconditioning” induced by physical exercise and detected in both humans and animals (Marongiu and Crisafulli, 2014). This phenomenon consists of ischemia short episodes that render the myocardium more resistant to subsequent more prolonged ischemic events. In addition, HIF-1α has been discovered to activate the downstream genes of VEGF such as inducible nitric oxide synthase (iNOS) in cardiomyocytes and endothelial cells, resulting in an increase in angiogenesis and cardioprotection (Jung et al., 2000; Tekin et al., 2010).

In the past two decades, Anversa et al. discovered that c-kit–positive cardiac stem cells and hematopoietic stem cells derived from the bone marrow can regenerate the heart muscle following a myocardial infarction (Beltrami et al., 2001; Anversa et al., 2013). According to these studies, by injecting these stem cells into the heart, they could differentiate into cardiomyocytes and coronary vessels, leading to tissue repair. This result, whether it had been true, would have had a decisive relief for a very large number of patients all over the world. However, several of these studies were recently retracted from prestigious journals, and Harvard Medical School and Brigham and Women’s Hospital have recommended the retraction of other 31 papers of Anversa’s group (Drazen, 2018; Dyer, 2018). Therefore, the research of physical exercise-related stimuli inducing a cardioprotective phenotype results to be really important for preventing cardiovascular diseases and improving heart function following injury. To this regard, little is known about the signaling molecules that regulate capillary growth in healthy myocardium according to the exercise intensity. This limitation might be due to controversial evidence about the stimulation of new capillaries in healthy hearts by physical exercise and might depend on the intensity and length of exercise training selected for analyzing cardiac microvasculature (Brown, 2003). It has been indeed reported that exercise-induced formation of new capillaries is transient as capillaries transform into arterioles (Brown, 2003). In previous studies, we showed a significantly increased capillary proliferation in mouse hearts following 30 and 45 days of an endurance training protocol (Bellafiore et al., 2007, 2013). Therefore, the aim of the present study was to investigate the localization and expression pattern of VEGFR-1/Flt-1, VEGFR-2/Flk-1, HIF-1α, and iNOS in the angiogenesis of mouse hearts in response to mild, moderate, and high intensity of endurance training. We speculated response patterns that differ among examined molecules and depend on the training workload.

## Materials and Methods

### Experimental Design

Sixty-three male 10-week-old Swiss mice were randomly divided into seven groups. Four groups were selected as sedentary controls (C) (0, 15, 30, and 45 days) and three groups were trained through an endurance protocol for 15 (T15), 30 (T30), and 45 days (T45). Mice were trained for 5 days/week on a rotating treadmill progressively increasing both workload intensity and training time (Di Felice et al., 2007). The exercise training intensity performed by T15, T30, and T45 mice corresponded to a mild, moderate, and high intensity, respectively. The study conforms to the *Guide for the Care and Use of Laboratory Animals* (NIH Publication No. 85-23, revised 1996) and was approved by the local Ethical Committee of Palermo University (Comitato Etico Palermo 1). We carried out this study using the same samples of heart prepared in our previous studies, in which we detected left ventricle hypertrophy associated with a significant increase in the capillary proliferation after 30 and 45 days of endurance training (Bellafiore et al., 2007, 2013).

### Immunostaining Analyses

In order to examine the localization and expression of VEGFR-1/Flt-1, VEGFR-2/Flk-1, HIF-1α, and iNOS, immunohistochemical analyses were performed. Hearts were fixed with formalin, embedded with paraffin, and cut to obtain 5 μm sections. After incubation of sections for 10 min with 0.3% H_2_O_2_, a serum-free protein block (DAKO, Carpinteria, USA) was added for 10 min. Sections were then incubated with the primary antibodies against VEGFR-1/Flt-1 (1:200; Chemicon^®^ International, Serological Company, EU), VEGFR-2/Flk-1 (1:100; Chemicon^®^ International, Serological Company, EU), HIF-1α (1:100; Chemicon^®^ International, Serological Company, EU), and iNOS (1:50; Calbiochem^®^, San Diego, CA, USA) for 1 h at room temperature (RT). Non-immune mouse serum was substituted for negative controls. Before adding the primary antibodies for HIF-1α, the slides were treated with monohydrated citrate buffer (pH 6.0, 0.01 M) in a water bath for 40 and 10 min, respectively, at 100°C for the antigen retrieval. After incubation for 10 min with a biotinylated secondary antibody, AEC chromogen (DAKO, Carpinteria, CA, USA) was used to develop the horseradish peroxidase (HRP)-streptavidin complex.

### Western Blotting

The expression levels of VEGFR-1/FLT-1, VEGFR-2/FLK-1, HIF-1α, and iNOS were measured in the left ventricle (LV) from each single mouse of the control and trained groups by immunoblot analyses. Frozen LV fragments were homogenized in a lysis buffer, containing protease inhibitors, and centrifuged at 13,000 rpm for 10 min at 4°C. Total cellular lysate fraction was collected, and protein concentration was determined using a colorimetric assay (Bio-4 Rad, Philadelphia, USA). About 20 μg of protein samples for each lane and a protein marker (Bio-Rad, Philadelphia, USA) were separated by 8% SDS-PAGE and transferred to a nitrocellulose membrane. After 1 h at RT with a blocking buffer, each membrane was incubated with primary antibodies against VEGFR-1/Flt-1 (1:1000; Chemicon^®^ International, Serological Company, EU), VEGFR-2/Flk-1 (1:500; Chemicon^®^ International, Serological Company, EU), HIF-1α (1:500; Chemicon^®^ International, Serological Company, EU), and iNOS (1:500; Calbiochem^®^, San Diego, CA, USA) overnight at 4°C. After washing, each membrane was incubated with HRP-conjugated secondary antibody for 1 h at RT, and signals were detected using an enhanced chemiluminescence (ECL, Amersham Bioscience, UK) for autoradiography. After stripping, the same membranes were incubated with β-actin antibody (1:1000; Sigma-Aldrich, USA), and the ratio of proteins of interest and β-actin was determined. The intensity of bands was quantified by computer-assisted image analysis (ImageJ, Media Cybernetics, Silver Spring, MD, USA) calculating pixel number per cm^2^.

### Statistical Analysis

Data are expressed as means ± standard deviations. One-way ANOVA test with Bonferroni’s multiple comparison test was used to analyze significant differences. Values were considered significantly different at *p* < 0.05.

## Results

### Cardiac Expression of VEGFR-1/Flt-1 and VEGFR-2/Flk-1 in Response to Physical Exercise

**Figure 1:**
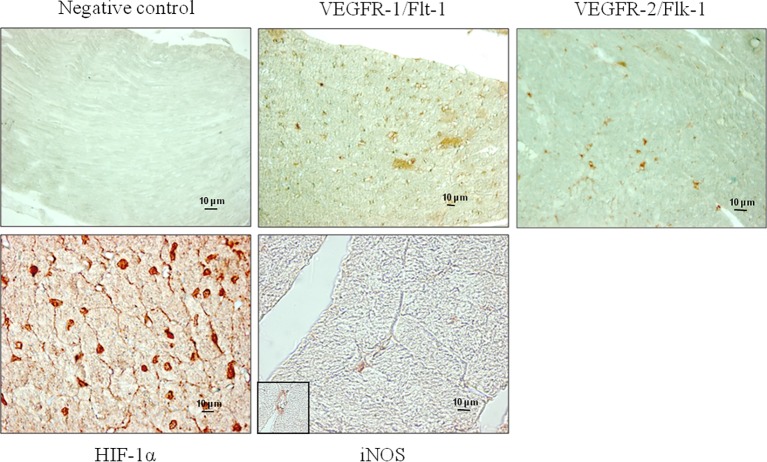
Representative microphotographs show the localization of VEGFR-1/Flt-1, VEGFR-2/Flk-1, HIF-1α, and iNOS performed by immunohistochemical analyses in the myocardium of T15 mice. Non-immune mouse serum was substituted for the negative controls. The specificity of the antibodies used was confirmed by the absence of staining in the negative controls.

**Figure 2:**
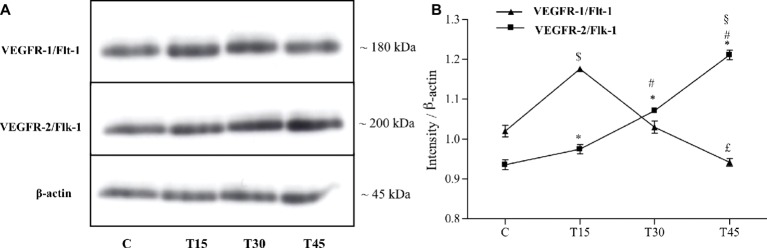
Western blotting **(A)** and quantitative analysis **(B)** to measure VEGFR-1/Flt-1 and VEGFR-2/Flk-1 expression in hearts from trained and control mice. **(B)** VEGFR-1/Flt-1 protein levels resulted significantly higher in T15 than in groups C, T30, and T45. Conversely, T45 animals showed a lower VEGFR-1/Flt-1 expression than C and T30 mice ($*p* < 0.05 T15 vs. T30 and T45; £*p* < 0.05 T45 vs. C and T30). VEGFR-2/Flk-1 expression gradually increased in trained mice (T15, T30, and T45) than control ones. Moreover, VEGFR-2/Flk-1 expression was higher in groups T30 and T45 than T15 ones and in T45 mice than T30 ones (*p < 0.05, T15, T30 and T45 vs. C; #*p* < 0.05, T30 and T45 vs. T15; §*p* < 0.05,T45 vs. T30).

Both receptors, VEGFR-1/Flt-1 and VEGFR-2/Flk-1, were expressed in subepicardial and subendocardial regions and specifically localized in the vascular endothelial cells as shown in Figure 1. We observed the same localization in the hearts from both control and trained mice (data not shown). From immunoblotting analyses, we obtained a band with molecular weight of about 180 kDa corresponding to VEGFR-1/Flt-1 protein (Figure 2A) as reported in the literature. Because samples from C, T15, T30, and T45 groups did not significantly differ within the same group, a representative sample from each group has shown (Figures 2A,B). The expression of VEGFR-1/Flt-1 was higher in T15 mice than in C, T30, and T45 groups (T15: 1.17 ± 0.005 vs. C: 1.02 ± 0.01, T30: 1.03 ± 0.01, and T45: 0.94 ± 0.01; *p* < 0.05; Figure 2B). T30 animals did not show any significant difference compared with C groups. Moreover, in T45 animals, VEGFR-1/Flt-1 expression was lower than C and T30 mice (T45: 0.94 ± 0.01 vs. C: 1.02 ± 0.01 and T30: 1.03 ± 0.01; *p* < 0.05; Figure 2B).

As regards to VEGFR-2/Flk-1 expression, we found a band with molecular weight of about 200 kDa that gradually increased in trained mice (T15, T30, T45) compared to controls (C) (T15: 0.97 ± 0.01, T30: 1.07 ± 0.007, and T45: 1.21 ± 0.01 vs. C: 0.93 ± 0.01; *p* < 0.05). In particular, VEGFR-2/Flk-1 expression was higher in groups T30 and T45 than in T15 (T30: 1.07 ± 0.007 and T45: 1.21 ± 0.01 vs. T15: 0.97 ± 0.01; *p* < 0.05) and in T45 rather than T30 mice (T45: 1.21 ± 0.01 vs. T30: 1.07 ± 0.007; *p* < 0.05; Figure 2).

### Regulation of HIF-1α Expression by the Endurance Training in the Myocardium

**Figure 3:**
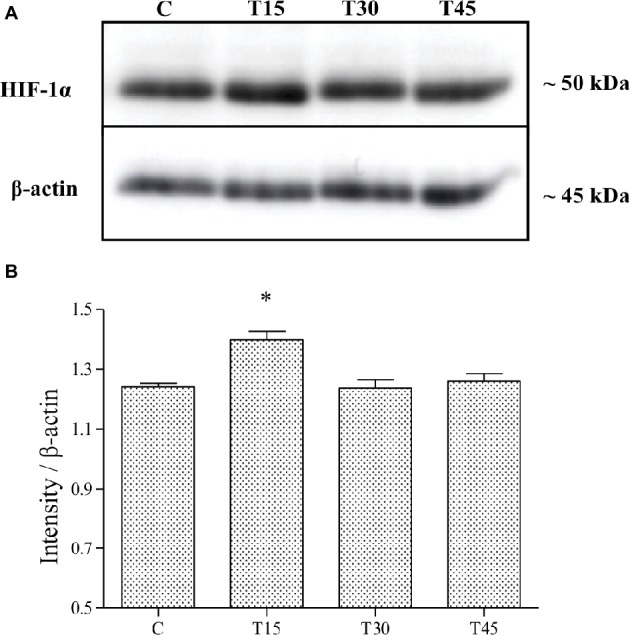
HIF-1α expression was evaluated by Western blotting analysis **(A)** in hearts from trained and control mice and its intensity measured with an image software **(B)**. HIF-1α levels resulted significantly higher in T15 than in C, T30, and T45 groups (*p < 0.05, T15 vs. C, T30, and T45).

Immunostaining analyses showed that HIF-1α protein was specifically localized in the nucleus of vascular endothelial and myocardial cells of control and trained animals (Figure 1). The expression of HIF-1α was confirmed by immunoblotting analyses (Figure 3A), which showed a band with molecular weight of about 120 kDa. As samples from C, T15, T30, and T45 did not significantly differ within the same group, a representative sample from each group is shown (Figure 3). As shown in Figure 3B, it is evident that HIF-1α expression was significantly higher in T15 animals than in T30 and T45 control mice (T15: 1.40 ± 0.11 vs. C: 1.24 ± 0.05, T30: 1.24 ± 0.12, and T45: 1.26 ± 0.10; *p* < 0.05). Moreover, control groups, T30, and T45 showed the same expression (Figure 3B).

### Analysis of iNOS Cardiac Expression Related to Physical Exercise

**Figure 4:**
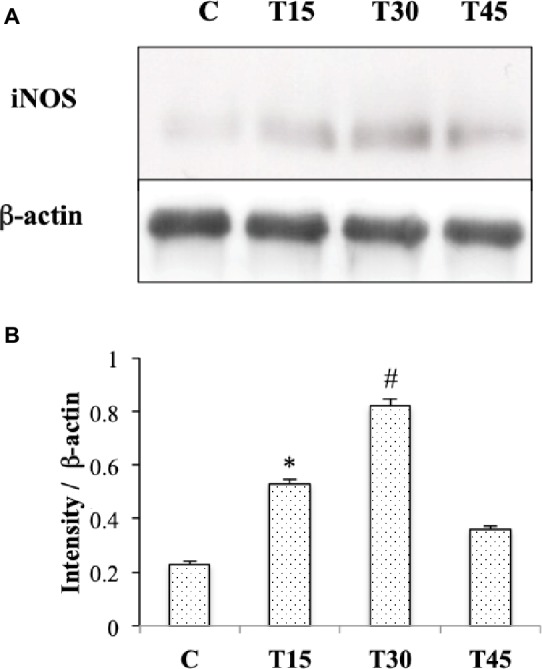
Western blotting **(A)** and quantitative analyses **(B)** were performed to measure iNOS expression in hearts from trained and control mice. iNOS protein levels were significantly higher in T15 and in T30 than in control and T45 mice (*p < 0.05,T15 vs. C and T45; #*p* < 0.05,T30 vs. C and T45).

In Figure 1, we observe that iNOS protein was expressed in the cells of interstitial connective tissue and smooth muscle of the hearts from T30 mice. The same localization was found in hearts from T15, T45, and control animals (data not shown). Evaluation of iNOS expression (Figures 4A,B) showed a significantly higher level in T15 and T30 groups than in C and T45 animals (T15: 0.48 ± 0.01 and T30: 0.92 ± 0.02 vs. C: 0.23 ± 0.009 and T45: 0.36 ± 0.01). Furthermore, no significant difference was detected between T45 and C animals. As samples from C, T15, T30, and T45 did not significantly differ within the same group, a representative sample from each group is shown (Figure 4).

## Discussion

In this study, we examined the relationship between VEGFR-1/Flt-1, VEGFR-2/Flk-1, HIF-1α, iNOS expression and angiogenesis in heart in response to low, moderate, and high exercise intensity. To this end, we carried out the analyses in myocardial tissue samples in which we previously found a relevant increase in the capillary proliferation following 30 and 45 days of moderate and high intensity endurance training, respectively (Bellafiore et al., 2007, 2013). The main findings in the present study were that the expression levels of the examined molecules were dissimilar according to the neocapillarization extent and physical exercise intensity. Human epidemiological and animal studies have shown that a moderate to high intensity exercise is the best quantity that results in a reduction in the cardiovascular risk (Alleman et al., 2015). A recent paper reported that high intensity treadmill exercise training for up to 4 weeks produced greater increases in new capillary formation than low intensity exercise training in rat hearts (Waring et al., 2014). However, results concerning the regulation of VEGFR-1/Flt-1, VEGFR-2/Flk-1, HIF-1α, and iNOS expression by exercise different intensities in healthy heart angiogenesis are limited.

Several studies reported an increase in VEGFR-1/Flt-1 and VEGFR-2/Flk-1 expression after 8 weeks of swim training or 10 weeks of treadmill running in rat heart angiogenesis (Iemitsu et al., 2006; Marini et al., 2008). In our study, VEGFR-1/Flt-1 and VEGFR-2/Flk-1 proteins were specifically expressed in the vascular endothelial cells of myocardium and differently regulated by intensity and length of the endurance training. In particular, VEGFR-1/Flt-1 expression increased in T15 mice and reduced in T30 and T45 animals exhibiting the same level as the controls. On the other hand, VEGFR-2/Flk-1 expression gradually increased in mice trained for 15, 30, and 45 days. This dissimilar pattern of expression suggests different roles of these receptors in the angiogenesis process such as also reported by Shibuya (2006). According to this author, VEGFR-1/Flt-1 is a negative regulator for angiogenesis during embryogenesis, while it stimulates inflammation, tumor growth, and metastasis in adulthood. By contrast, VEGFR-2/Flk-1 transduces the major signals for angiogenesis. In effect, in old age myocardium, this receptor promotes an angiogenic cascade through phosphorylation of Akt and activation of endothelial nitric oxide (eNOS) pathway in response to exercise training (Iemitsu et al., 2006). Furthermore, Milkiewicz et al. (2003) showed a good spatial and temporal correlation between VEGFR2/Flk-1 protein levels and capillary growth induced by muscle activity. These findings are in agreement with our data that showed a similar pattern between VEGFR-2/Flk-1 expression and capillary proliferation in hearts of trained mice suggesting receptor’s involvement in the proliferation of vascular endothelial cells in response to endurance exercise (Bellafiore et al., 2007, 2013).

The upregulation of VEGFR-1/Flt-1 after 15 days of exercise training might be explained by the request of this factor in the early steps of angiogenic sprouting when endothelial cell migration rather than cell proliferation is necessary. This hypothesis is supported by the same expression pattern between VEGFR-1/Flt-1 and MMP-9 pro-enzyme in exercise-related cardiac angiogenesis (Bellafiore et al., 2013). Moreover, Waltenberger et al. (1994) showed that the activation of VEGFR-2/Flk-1 receptor by VEGF in cells devoid of VEGFR-1/Flt-1 resulted in a mitogenic response, while the activation of VEGFR-1/Flt-1 by VEGF did not induce cell proliferation in cells lacking VEGFR-2/Flk-1.

Hypoxia has been demonstrated to be a potent stimulus for the activation of HIF-1α protein generating capillary growth in heart tissue (Shweiki et al., 1992; Brown, 2003; Prior et al., 2004) through the activation of VEGF pathway genes, including VEGFR-1/Flt-1 but not VEGFR-2/Flk-1 (Gerber et al., 1997). In our study, we detected that HIF-1α was specifically expressed in the nucleus of vascular endothelial cells and myocardiocytes from both sedentary and trained mouse hearts. The expression of this protein under normoxic conditions was confirmed in the myocardium also by other authors (Stroka et al., 2001; Giusti et al., 2009). This finding might be due to a basal induction of genes that are necessary to providing cellular energy requirements. We found that HIF-1α expression significantly increased in mice trained for 15 days and reduced in animals trained for 30 and 45 days reaching the same level as the controls. The reduced expression in T30 and T45 animals might be explained by an increase in the supply of blood flow and O_2_ in the heart as a consequence of an increase in the capillary area, as shown in our previous study (Bellafiore et al., 2007). On the contrary, in the hearts of T15 mice that did not show a significantly increased capillary area, brief and transient episodes of hypoxia might occur during the cardiac cycle in response to exercise and determine the upregulation of HIF-1α as shown in other studies (Stroka et al., 2001; Brown, 2003; Marini et al., 2008; Giusti et al., 2009). The increased HIF-1α expression is indicative for an involvement of hypoxia signaling in the exercise-induced upregulation of VEGF receptors. Comparing the expression of HIF-1α and VEGFR-1/Flt-1, we noted that their patterns are similar postulating an upregulation of VEGFR-1 by HIF-1α after 15 days of endurance training in agreement with Gerber et al. (1997).

Nitric oxide (NO) as well as hypoxia has been reported to upregulate VEGF gene by enhancing HIF-1α activity and significantly contributing to the prosurvival/proangiogenic program of capillary endothelium (Ziche and Morbidelli, 2000). Indeed, intermittent hypoxia has been described to induce protective effects against myocardial infarction in rodents *via* a signaling mechanism that depends upon iNOS (Tekin et al., 2010). Furthermore, Akita et al. (2007) showed that an increased expression of iNOS by eNOS upregulation was associated with late cardioprotection against ischemia-reperfusion injury in mouse hearts after 7 days of treadmill exercise at a 60–70% maximal oxygen uptake. In our study, iNOS cardiac expression was higher than corresponding controls in response to mild and moderate exercise intensity, and in our best knowledge, this is the first study that reports the involvement of iNOS in healthy heart neocapillarization according to the exercise intensity.

In the light of the increased expression of iNOS and HIF-1α in T15 hearts, we propose iNOS upregulation by HIF-1α protein in the angiogenic process in agreement with the study of Jung et al. (2000). In fact, these authors exhibited that HIF-1α-binding site was required for transcriptional activation of iNOS gene in rat cardiomyocytes under hypoxic conditions. In our study, the localization of HIF1α and iNOS in different cell types suggests a cross talk among myocardium, vasculature, and connective tissue to promote capillary growth related to exercise in a paracrine fashion.

NO also participates in the regulation of VEGFR-1/flt-1 gene in response to exercise as shown by NOS inhibition that reduced the exercise-induced increase in VEGFR-1/flt-1 mRNA in rat skeletal muscle (Gavin and Wagner, 2002). Therefore, in our experimental model, the upregulation of iNOS, HIF-1α, and VEGFR-1/flt-1 in T15 mouse hearts suggests the involvement of iNOS in the angiogenesis process related to endurance training.

A limitation of this study is the lack of data coming from the expression of circulating angiogenic factors produced by other organs that could be associated with the exercise-related adaptive effects of cardiac muscle. The release of circulating factors by the exercising skeletal muscle might have a role in supporting angiogenesis of healthy or pathological hearts for prevention and rehabilitation of heart failure (Di Raimondo et al., 2017). Recently, it has been reported that skeletal muscle cells communicate with heart cells in response to contraction or exercise training through the endocrine secretion of myokines (Giudice and Taylor, 2017). In particular, plasma levels of follistatin-like 1 (FSTL-1) glycoprotein increased after strength training (Norheim et al., 2011). Moreover, the administration of this myokine caused an increase in the number of dividing cardiomyocytes and angiogenesis in the peri-infarct region of mouse and swine heart (Wei et al., 2015). On the contrary, systemic levels of VEGF were frequently unaltered in response to exercise, pointing toward mainly local effects (Landers-Ramos et al., 2014).

Although the promising results of Anversa’s studies on the application of stem cells for the repair of the infarcted heart region have been retracted, the activation of resident or circulating stem/precursor cells by physical exercise might induce a cardioprotective phenotype against insults (Bellafiore et al., 2006). Indeed, recently, an increase in number and activity state of these cells was discovered in exercised hearts, resulting in an amplified expression of transcription factors involved in the differentiation toward either the cardiomyocyte or capillary lineages. Moreover, these adaptations have been observed to be dependent on exercise duration and intensity (Waring et al., 2014). In skeletal muscle, the number of satellite cells is positively correlated to the capillarization of the myofiber, and angiogenesis results to be essential for muscle repair (Mounier et al., 2011). Therefore, increasing knowledge about molecular effectors of cardiac capillary growth related to exercise can be relevant in pre-clinical and clinical studies to generate native and synthetic biomaterials or three-dimensional structures able to induce stem cell differentiation and which might be used in pre-clinical and clinical studies (Di Felice et al., 2015).

In conclusion, the present study shows that VEGFR-1/Flt-1, VEGFR-2/Flk-1, HIF-1α, and iNOS are involved in the cardiac angiogenesis, and their expression is regulated by the intensity of endurance training. In detail, the expression of VEGFR-1/Flt-1 and HIF-1α is upregulated by an exercise mild intensity, while VEGFR-2/Flk-1 level progressively enhances with increasing workload. Differently, iNOS protein is modulated by a moderate intensity exercise. In the light of our results, we retain HIF-1α as a central regulator of exercise-induced angiogenesis pathway in the myocardium. HIF-1α might act as an upstream regulator of VEGFR-1 and iNOS in modulating cell proliferation and vascular relaxation. VEGF pathway appears to be involved in this regulation mechanism, and VEGF might act on heart endothelial cells in a paracrine and endocrine manner. Our endurance protocol was formulated for training human subjects and adapted to mice according to their body weight; therefore, understanding the molecular basis for exercise-induced angiogenesis is important in developing exercise strategies to protect the heart by insults.

## Data Availability

All datasets generated for this study are included in the manuscript and/or the supplementary files.

## Author Contributions

MB was responsible for study design, interpretation of data, and draft of manuscript. GB carried out data acquisition and participated in conceiving of the study and drafting manuscript. AB participated in exercise training sessions. AP participated in conceiving of the study and drafting manuscript. All authors read and approved the final manuscript.

### Conflict of Interest Statement

The authors declare that the research was conducted in the absence of any commercial or financial relationships that could be construed as a potential conflict of interest.
